# The “Funny” Current (I_f_) Inhibition by Ivabradine at Membrane Potentials Encompassing Spontaneous Depolarization in Pacemaker Cells

**DOI:** 10.3390/molecules17078241

**Published:** 2012-07-09

**Authors:** Yael Yaniv, Victor A. Maltsev, Bruce D. Ziman, Harold A. Spurgeon, Edward G. Lakatta

**Affiliations:** Laboratory of Cardiovascular Science, Intramural Research Program, National Institute on Aging, NIH, 5600 Nathan Shock Dr., Baltimore, MD 21224, USA

**Keywords:** pacemaker cell automaticity, pacemaker Ca^2+^ clock, arrhythmia

## Abstract

Recent clinical trials have shown that ivabradine (IVA), a drug that inhibits the funny current (*I_f_*) in isolated sinoatrial nodal cells (SANC), decreases heart rate and reduces morbidity and mortality in patients with cardiovascular diseases. While IVA inhibits *I_f_*, this effect has been reported at essentially unphysiological voltages, *i.e.*, those more negative than the spontaneous diastolic depolarization (DD) between action potentials (APs). We tested the relative potency of IVA to block *I_f_* over a wide range of membrane potentials, including those that encompass DD governing to the SANC spontaneous firing rate. A clinically relevant IVA concentration of 3 μM to single, isolated rabbit SANC slowed the spontaneous AP firing rate by 15%. During voltage clamp the maximal *I_f_* was 18 ± 3 pA/pF (at −120 mV) and the maximal *I_f_* reduction by IVA was 60 ± 8% observed at −92 ± 4 mV. At the maximal diastolic depolarization (~−60 mV) *I_f_* amplitude was only −2.9 ± 0.4 pA/pF, and was reduced by only 41 ± 6% by IVA. Thus, *I_f_* amplitude and its inhibition by IVA at physiologically relevant membrane potentials are substantially less than that at unphysiological (hyperpolarized) membrane potentials. This novel finding more accurately describes how IVA affects SANC function and is of direct relevance to numerical modeling of SANC automaticity.

## 1. Introduction

Heart rate is a primary determinant of cardiac output, an index of myocardial work and myocardial oxygen demand, an index of ATP production rate. An elevation of resting heart rate, therefore, increases the oxygen demand and when the energy reserve capacity is limited can promote an imbalance between energy demand and supply. Clinical evidence has shown that an increase in heart rate in patients with ischemic heart diseases is associated with an increased cardiovascular morbidity and mortality (e.g., [1,2]), and that drugs which reduce the heart rate can improve myocardial pumping performance and energy balance efficiency. Recent clinical trials (for a review cf. [3]) have shown that administration of ivabradine (IVA), a drug that inhibits *I_f_*, *i.e.*, an inward current activated by hyperpolarization of the cell membrane, is associated with a reduction in heart rate and reduction in morbidity and mortality in patients with cardiovascular diseases.

At concentrations that are achieved by approximately clinical doses, IVA in isolated rabbit SANC *in vitro*, inhibits *I_f_* and has no significant action on other membrane ion channels [4], and has no direct effect on myocardial contractility [5]. The relative reduction in *I_f_ in vitro* by IVA, however, has been reported only at hyperpolarized membrane potentials, *i.e.*, those more negative to the maximum diastolic potential (MDP), far from the physiological voltages over which *I_f_* can contribute to spontaneous diastolic depolarization (DD) of the surface membrane in rabbit SANC [4,6]. Thus the relative reduction in *I_f_* by IVA over a physiological voltage range is unknown. On the other hand, inhibition of funny-channels by IVA occurs only when they are open [4], *i.e.*, when *I_f_* is activated. Since *I_f_* is activated less at physiological potentials than at hyperpolarized (unphysiological) potentials, we hypothesize that the relative blockade of *I_f_* by IVA is voltage dependent, and may be less at physiologically relevant membrane potentials than at hyperpolarized potentials which have previously been studied. If this were the case, it would indeed be of direct relevance to pacemaker cell normal automaticity as it would allow a more precise portrayal of *I_f_* and its inhibition by IVA in numerical models of the coupled-clock system that regulates normal automaticity.

We found by measuring AP and ionic currents in single isolated SANC, that the IVA blockade is, indeed, less pronounced at physiological membrane potentials, *i.e.*, those encompassing DD (~−60 mV) than at potentials more negative to the MDP.

## 2. Results

### 2.1. IVA Blocks I_f_ in SANC

**Figure 1:**
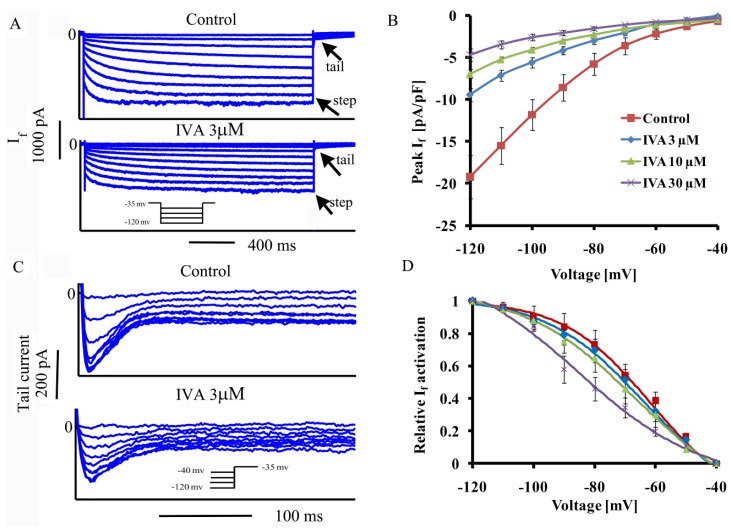
Effects of IVA on amplitude and activation kinetics over a wide range of membrane potential range. (**A**) Representative *I_f_* traces in SANC recorded before and after application of IVA and (**B**) average peak *I_f_* amplitude-voltage relationship; (**C**) representative *I_f_* tail traces recorded in SANC before and after application of IVA and (**D**) *I_f_* steady-state activation curve under control conditions and in the presence of different concentrations of IVA (n = 9 for each concentration).

Representative examples of *I_f_* as a function of membrane potential during voltage clamp with and without 3 μM IVA are shown in Figure 1A. The average effect of IVA (n = 9 for each IVA concentration) on the I–V relationship of peak *I_f_* is shown in Figure 1B. *I_f_* activation was calculated from the peak tail current 5 min after IVA application (at steady state of blockade). *I_f_* tail density, at each membrane potential is expressed relative to its maximal value at −120 mV in control. The average *I_f_* characteristics in the presence or absence of IVA are summarized in Table 1. On average (Figure 1D) at low concentration (3 μM), IVA decreased the peak *I_f_* without significantly shifting its activation curve (Table 1), but at higher concentrations of 10 and 30 μM, IVA shifted the activation curve, *i.e.*, V_1/2_ was shifted to lower potentials from −68 ± 4 to −74 ± 4 and −77 ± 5 mV, respectively.

**Table 1:** *I_f_* characteristics,* *p* < 0.05 *vs.* control, ^#^*p* < 0.05 *vs.* ivabradine 3 μM.

	Control	Ivabradine	Ivabradine	Ivabradine
		3 μM	10 μM	30 μM
		(n = 9)	(n = 9)	(n = 9)
**V_1/2_ activation (mV)**	−68 ± 4	−70 ± 4	−74 ± 4 *	−77 ± 5 *
		(*p* = 0.07)	(*p* = 0.02)	(*p* = 0.04)
**Max ** **τ_act_**	157 ± 11	120 ± 17	106 ± 17 *	80 ± 13 *
**(ms)**		(P = 0.5)	(P = 0.05)	(P = 0.02)
***I_f_* density (pA/pF) **	4.5 ± 0.7	2.1 ± 0.3 *	1.6 ± 0.2 *	1.1 ± 0.2 *^,#^
**(V = control V_1/2_ activation) **		(P = 0.04)	(P = 0.008)	(0.004)
***I_f_* (pA/pF) (V = MDP) **	3 ± 0.43	2 ± 0.3 *	1.5 ± 0.2 *	1.3 ± 0.2 *
		(P = 0.045)	(P = 0.04)	(P = 0.01)

**Figure 2:**
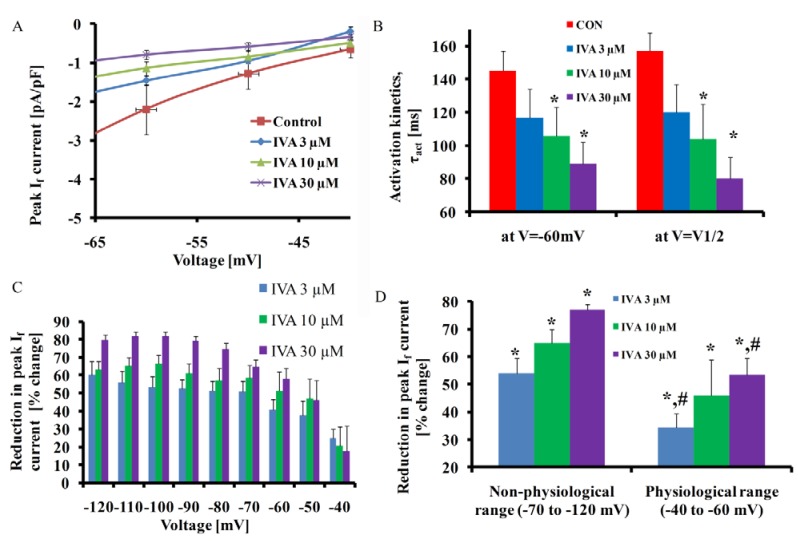
Effects of IVA on *I_f_* amplitude and activation kinetics in the physiological membrane potential range. (**A**) Average peak *I_f_* amplitude-voltage relationship in physiologicaly range of membrane potential; (**B**) *I_f_* activation kinetics at physiologic and non-physiologic membrane potentials; (**C**–**D**) reduction in *I_f _* amplitude over wide range of membrane potentials including physiological and non-physiological values (n = 9 for each concentration). * *p* < 0.05 *vs.* control, ^#^ *p* < 0.05 *vs.* non-physiological range.

On average, at V_1/2_, IVA at 3, 10 and 30 μM IVA (n = 9 for each concentration) decreased the peak *I_f_* by 49 ± 6, 68 ± 4, 77 ± 10 percent control, respectively. While the concept of V_1/2_ is instructive in biophysical terms, *I_f_* V_1/2_ occurs at an unphysiological membrane potential, *i.e.*, outside the range over which the membrane potential in rabbit SANC spontaneously cycles (from −65 to +30 mV) during spontaneous AP firing at a rate of ~2.5 Hz. Figure 2A illustrates peak *I_f_* as a function of membrane potential over the entire range of DD where *I_f_* has physiological importance by contributing to pacemaker function. Note that *I_f_* amplitude, even in control is small over this voltage range. Figure 2B shows that activation kinetics, expressed as τ_act_, at −60 mV and V_1/2_, are smaller at larger IVA concentrations. Figure 2C shows the voltage dependence of the reduction in peak *I_f_* by IVA. Note that peak *I_f_* and relative IVA inhibition are higher at unphysiological membrane potentials: on average (n = 9 for each IVA concentration) steady-state blockade (95% interval) was achieved at −92 ± 4, −89 ± 5 and −88 ± 5 mV with 3, 10 and 30 μM IVA, respectively. At the physiological range of membrane potentials (−60 to −40 mV) average reductions in peak *I_f_* were only 34.5 ± 13, 46 ± 13 and 53.5 ± 6% with 3, 10 and 30 μM IVA in contrast to 54 ± 5.5, 65 ± 5 and 77 ± 2% over the unphysiologic membrane potential range with 3, 10 and 30 μM IVA, respectively (Figure 2D).

### 2.2. Concentration-Dependent Block of I_Ca,L_ by IVA in SANC

**Figure 3:**
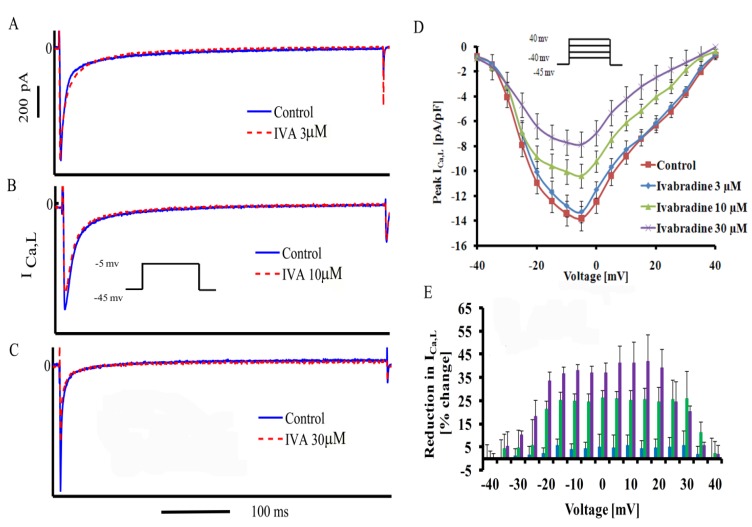
Effect of IVA on *I_ca,L_*. (**A**–**C**) Representative effect of IVA on maximal *I_ca,L_*; (**D**) average *I_Ca,L_* amplitude-voltage relationship under control conditions and in the presence of different IVA concentrations, and (**E**) relative reduction in *I_Ca,L_* as a function of membrane potential (n = 12 for control and 3 μM IVA; n = 9 for 10 and 30 μM IVA).

Concentrations of IVA higher than 3 μM are known also to affect *I_Ca,L_* [4]. We measured the effect of IVA on *I_Ca,L_* at the concentrations of IVA used to measure the IVA effects on *I_f_*. Representative examples of *I_Ca,L_* at a maximal peak current measured at −5 mV and average data of *I_Ca,L_* density as a function of membrane potential are presented in Figure 3A–D, respectively. Average *I_Ca,L_* characteristics are summarized in Table 2. In the presence of 3 μM IVA, peak *I_Ca,L_* decreased by 3.4 ± 1 percent from control (from −13.9 ± 1 to −13.3 ± 1 pA/pF, n = 12). This reduction, however, did not statistically differ from *I_Ca,L_* run-down (4.7 ± 1.5%, n = 9) in the absence of drug over the 5 min experimental period. At higher concentrations (10 and 30 μM), IVA decreased peak *I_Ca,L_* by 22.4 ± 3% (to −9.9 ± 1.4 pA/pF, n = 9) and by 36 ± 3% (to −8.1 ± 1 pA/pF, n = 9), respectively. Figure 3E shows the reduction in *I_Ca,L_* as function of the membrane potential. The relative IVA blockade of *I_Ca,L_* was maximal at maximal *I_Ca,L_* (V = −5 mV).

**Table 2:** I_Ca,L_ characteristics, * *p* < 0.05 *vs.* control, ^#^ *p* < 0.05 *vs.* IVA 3 μM.

	Control	Ivabradine	Ivabradine	Ivabradine
		3 μM	10 μM	30 μM
		(n = 12)	(n = 9)	(n = 9)
**Peak I_Ca,L_ density (pA/pF) **	13.9 ± 1	13.2 ± 1	9.85 ± 1.4 *	8.1 ± 1 *^,#^
		(*p* = 0.953)	(*p* = 0.0003)	(*p* < 0.001)
**Voltage at peak I_Ca,L_ (mV) **	−5.55 ± 1	−5.4 ± 0.7	−5 ± 0.1	−6 ± 0.8
		(*p* = 0.84)	(*p* = 1)	(*p* = 0.98)

### 2.3. IVA Reduces the SANC Spontaneous AP Firing Rate

**Figure 4:**
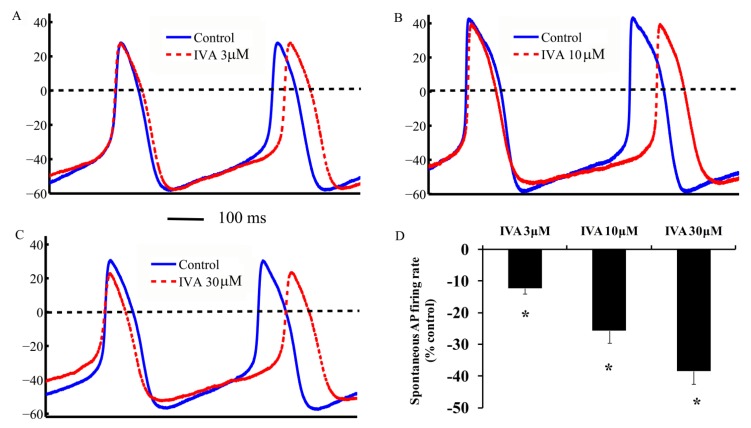
Effect of IVA on spontaneous AP firing rate. (**A**–**C**) Representative AP recordings and (**D**) average changes in the rate of AP-induced contractions in the presence of IVA (n = 10 for each concentration). * *p* < 0.05 *vs.* drug control.

To determine IVA effect on AP firing rate, we superfused IVA onto single SANC generating spontaneous APs. Representative examples of APs and average AP firing rate are presented in Figure 4A–C and average results for AP-induced contraction rate are presented in Figure 4D. The AP-firing rate on average (n = 10 for each concentration) was reduced by 13 ± 2% (from 175 ± 2 to 155 ± 9 beats/min), 26 ± 4% (to 133 ± 12 beats/min) and 39 ± 4% (to 110 ± 11 beats/min) by 3, 10 and 30 μM IVA, respectively. Note, in control experiments the time-dependent decrease in beating rate was 3 ± 1.5%.

### 2.4. Correlations Among I_f_, I_Ca,L_ and Spontaneous AP Cycle Length in the Absence and Presence of IVA

**Figure 5:**
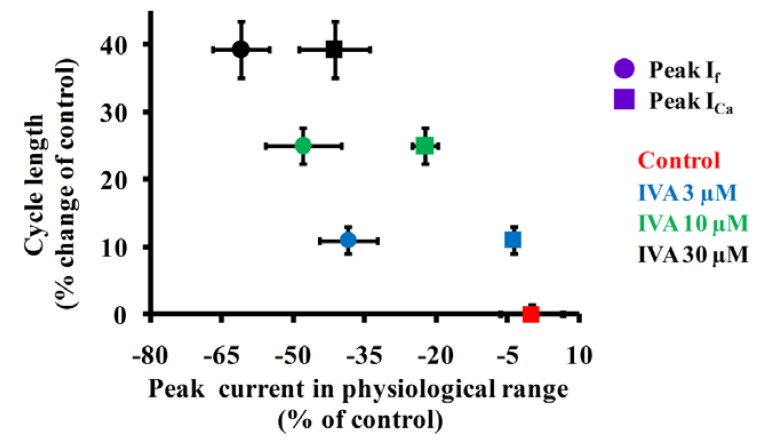
Relationships of AP firing rate as a function of *I_f_*, *I_Ca,L_* and AP firing rate. Effect of IVA on the relationship between spontaneous AP cycle length and peak *I_f_* and *I_Ca,L_* in physiological range of membrane potentials.

Figure 5 compares effects of IVA at three concentrations on cycle length as a function of relative reductions of maximal *I_Ca,L_* peak or *I_f_* peak over the physiological range of membrane potentials. A similar concentration dependent block of *I_f_* by IVA (the IVA concentrations vary from 0.1 to 100 μM) and AP firing rate has been reported previously [4]. At low concentration of IVA (3 μM), *I_Ca,L_* insignificantly changed, therefore *I_Ca,L_* cannot explain the change in spontaneous AP cycle length at this concentration. However, at the two higher concentrations of IVA, *I_Ca,L_* is substantially reduced, as also shown previously [4], and at these high IVA concentrations the prolongation in spontaneous AP cycle length is related to reductions in both *I_f_* and *I_Ca,L_*.

## 3. Discussion

Because the physiological function of *I_f_* is linked to its contribution to the diastolic depolarization, it is important to characterize the maximal *I_f_* and its activation kinetics within the physiologically relevant range, *i.e.*, above the MDP (~−60 mV in rabbit SANC). A novel finding of our study is that in SANC, the heart pacemaker cells, the relative IVA blockade of *I_f_* is voltage dependent. At voltages more negative than the MDP, both the *I_f_* amplitude and the *I_f_* blockade by IVA markedly exceed those encountered over the physiological range.

A comprehensive review of the literature on reported *I_f_* amplitude at the MDP compared to its maximal value (−120 mV) is presented in Table 3. Note that on average, *I_f_* amplitude at the MDP (~−60 mV) is only ~20% of the maximum *I_f_* (~−14 pA/pF) at −120 mV. Therefore current amplitude and effect of pharmacological interventions and specifically those of *I_f_* and IVA effect should be compared to the pre drug at normal membrane voltages which occur during AP firing, rather than those values measured at more negative, non-physiological voltages. In short, this approach is required to accurately interpret the results of biophysical measurements and pharmacological effects in physiologically relevant terms at physiological potentials and is also critical in the context of numerical modeling of pacemaker function.

**Table 3:** Biophysical properties of SAN *I_f_* in various species. MDP = Maximum diastolic potential. Values are estimated based on experimental data (from figures) in each of the listed papers.

Species	MDP [mV]	Amplitude at MDP [pA/pF]	Maximal Current at −120 mV [pA/pF]	Reference
Rabbit	−60	8	16	[7]
		10	30	[8]
		1	10	[9]
		1	5	[10]
		1	6	[11]
		1	18	[12]
		1	22	[13]
		4	20	[14]
		4	16	[15]
		3	19	[16]
		1	6	[17]
Mouse	−62	2	11	[18]
		2	22	[19]
		2	15	[20]
		1	20	[21]
		2	15	[22]
Dog	−58	1	6	[18]
		2	10	[23]
		2	11	[24]
Guinea pig	−61	1	12	[25]
Rat	−58	5	15	[26]
Cat	−68	3	6	[27]
Human	−62	1	8	[28]

Because the membrane potential *in vivo* spontaneously cycles within the physiological range, *i.e.*, about from −65 to 30 mV, we explored the reduction in AP firing rate by IVA under physiological conditions by measuring AP-induced contraction, or by utilizing a perforated patch clamp to measure spontaneous AP firing. At 3 μM, IVA reduced the spontaneous AP firing rate by 13 ± 2% control (Figure 2), similar to the reduction reported previously in rabbit SANC (16.2 ± 1.5% control) [29]. Not in order. Of note, in healthy human volunteers, a bolus of IVA reduces the heart rate during exercise by 10–15%, and this effect is sustained for 1 to 12 h [30]. Because IVA inhibition occurs on the inner cell membrane, and only when the *I_f_* channel is open [4], some prior studies have explored the effect of IVA on AP firing rate in SANC following an activation/deactivation protocol via a whole cell voltage clamp [6]. In this protocol, the membrane potential is set as low as to −100 mV every 6 s from a holding potential of −35 mV and then the recording configuration is switched to AP firing in current clamp. A greater reduction in AP firing rate by 3 μM IVA (~25%) has been observed in this non-physiological experimental protocol than in the present study [6].

At a clinically relevant concentration, IVA does not alter other cardiovascular functions (*i.e.*, blood pressure, cardiac electrophysiology, *etc.*) [31,32], and therefore it has a potential for therapeutic application by slowing the heart rate. We observed that at low concentrations (<3 μM) while IVA inhibits *I_f_* it does not affect *I_Ca,L_* in single isolated rabbit SANC (Figure 2). Similarly, Bois *et al.* [4] showed that low concentrations (<3 μM) IVA did not affect *I_Ca,L_* current (or *I_K_* current). Note that due to *I_Ca,L_* current run-down we cannot distinguish a drug effect that reduces *I_Ca,L_* less than 5%. However, our recent SANC coupled-clock numerical model [33] predicts that such a small decrease of 5% in *I_Ca,L_* without altering *I_f_* would reduce the spontaneous AP firing rate only by 2% (not shown). At higher IVA concentrations which inhibit *I_Ca,L_*, *I_Ca,L_* inhibition does not directly impact *I_f_* (because *I_f_* is inactivated at voltages lower than −40 mV); the decrease in *I_Ca,L_* however, reduces Ca^2+^ influx, and this has a substantial effect to reduce the AP firing rate by directly impacting the Ca^2+^ clock, and by indirectly reducing cAMP production. In this way *I_Ca,L_* inhibition by IVA indirectly impacts on *I_f_* current. L-type Ca^2+^ channel is constituted by a pore-forming α_1_ subunit. Four L-type α_1_-subumit have been cloned and classified in the Ca_v_1 gene family (for review cf. [34]). Recombinant and native Ca_v_1.3-mediated *I*_Ca,L_ displays a more negative activation and displays a more negative activation threshold than Ca_v_1.2. Of note, however, the lower activation threshold for Ca_v_1.3 *vs.* Ca_v_1.2 is still more positive to the voltage range where *I_f_* current becomes dominant in mice SANC [35]. Specifically, there is no documentation for a role for Ca_v_1.3 in mammalian pacemaker cells other than mice.

Aside from the effect of IVA to reduce the spontaneous AP firing rate of isolated SANC, additional perspectives regarding the role of *I_f_* in spontaneous AP firing rate of SANC require consideration. *I_f_* cannot be the sole mediator of diastolic depolarization, even under normal conditions, because (1) at MDP 54% reduction in *I_f_* (by 30 µM IVA, at MDP) mediates an apparent ~40% reduction in spontaneous AP firing rate and not a complete cessation of the heart rate; (2) The relative amplitude of *I_f_* during the diastolic depolarization is much smaller than *I_NCX_*, which has also been implicated as a major contributor to diastolic depolarization rate [18,36,37]. Because *I_f_* is larger in SANC from the periphery of the central SAN area than from its center where the AP impulse normally originated [9], and since the peripheral SANC have a lower MDP, the relative effect of IVA on *I_f_* may be stronger in SANC from the peripheral area. In this case, IVA effect would mostly affect the transition of AP to the atria, rather than having a major direct impact on the AP firing.

Although it is impossible to know the exact IVA concentration (diffusion, drug accumulation and *etc.*) achieved at the cellular level in patients, it has been estimated that the concentration is not higher than 3 µM [30]. During long treatment protocols in humans, IVA was reported to reduce mean resting heart rate by 11 to 22%, depending on the pre-drug heart rate [30,38]: The IVA-induced decrease in heart rate is higher at higher baseline heart rates. Note, however, that IVA is usually administered to patients in conjunction with a beta-blocker, which also can affect the heart rate [39]. The relative role of I_f_ may differ among species (Table 3), and this can bias the observed IVA effect on heart rate.

Recent observations in pacemaker cells provide strong evidence that normal automaticity in SANC is regulated by a coupled-clock system: a high basal (*i.e.*, in the absence of β-adrenergic receptor stimulation) Ca^2+^-activated adenylyl cyclase (AC) drives cAMP/protein kinase-A (PKA), and CaMKII-dependent protein phosphorylations of both surface membrane electrogenic proteins (“membrane clock”), and of sarcoplasmic reticulum (SR) proteins that generate rhythmic Ca^2+^ oscillations (“Ca^2+^ clock”) [18,36,37]. The degree of protein phosphorylation of the membrane and Ca^2+^ clocks regulates the coupling of the two-clocks, and the periodicity of the coupled clock controls the action potential (AP) rate, *i.e.*, generating the normal automaticity of SANC. Thus the bradycardic effect of IVA may not be interpreted to result soley from its effect to block *I_f_*, a membrane clock protein because the mutual membrane and Ca^2+^ clock environment and regulation strongly couple the function of both clocks. Changes in AP firing rate generated by a drug effect on the membrane-clock, in a feed-forward manner, will change intracellular Ca^2+^, and lead to further changes in the AP firing rate. Therefore, the net effect of IVA, even at the low concentration of 3 µM, on AP firing rate of the coupled-clock system may indeed be mediated by effects on both clocks: a direct effect on *I_f_* (a component of the “membrane-clock”) to regulate the AP firing rate, and an indirect effect on the Ca^2+^ clock (due to concomitant reduction in intracellular Ca^2+^ cycling due to clock coupling when the AP firing rate slows). Additional experiments are required to test this interesting possibility.

## 4. Experimental

### 4.1. Cell Preparation

Spontaneously beating sinoatrial node cells were isolated from New Zealand White rabbit hearts as previously described [40]. All animal experiments were approved by the Animal Care and Use Committee of the National Institutes of Health (protocol #034LCS2013). The dissociated cells were stored at 4 °C and were used within 10 h of isolation.

### 4.2. Electrophysiology and Cell Contraction

Spontaneous rhythmic cell contractions during spontaneous AP firing and APs were recorded to quantify the spontaneous AP firing rate in Tyrode solution at 35 ± 0.5 °C, contained the following (in mM): 140 NaCl, 5.4 KCl, 2 MgCl_2_, 5 HEPES, 1.8 CaCl_2_, and 5.5 glucose, and titrated to pH 7.4 with NaOH. The cell suspension was placed in a chamber on an inverted microscope and was allowed to settle for 20 min. Spontaneous cell contractions were measured as previously described [41]. Briefly, cells were imaged with an LSM-510 inverted confocal microscope using a 63x/1.4 N.A. oil immersion lens (Carl Zeiss, Oberkochen, Germany). Transmitted optics linescan images (using 633 nm He-Ne laser excitation, 512x1 pixels at 21.5 pixel/μm and 0.8 ms/line), were recorded with a scan line oriented along the short axis of the SANC. Spontaneous APs were recorded via a perforated patch-clamp with 35 µM β-escin (Sigma-Aldrich, St. Louis, MO, USA) added to the pipette solution that contained (in mM) the following: 120 K-gluconate, 2.5 NaCl, 2.5 MgATP, 2.5 Na_2_ATP, 5 HEPES and 20 KCl, and titrated to pH 7.2 with KOH. SANC contraction and AP measurements were recorded for 5 min under control conditions and 10 min following IVA application. 

### 4.3. If Measurements

*I_f_* was measured in whole cell patch clamp mode. Patch pipettes had a resistance of 2–4 MΩ and were filled with solution that contained the following (in mM): 100 K-gluconate, 2.5 MgATP, 2.5 Na_2_ATP, 5 HEPES, 20 KCl, 5 EGTA and 2 CaCl_2_ and titrated to pH 7.2 with KOH. Tyrode solution (as above) was used as extracellular solution. Membrane series resistance and whole cell and pipette capacities were routinely compensated electronically up to 90%. Voltage steps were applied for 2 s ranging from −120 to −40 mV in 10-mV increments from a holding potential of −35 mV. The voltage steps protocol was applied 3 min after the rupture patch and was repeated every minute for 5 min, following IVA application. The time course of *I_f_* activation (τ) was evaluated by fitting a monoexponential equation with the Clampfit program (Molecular devices): *I* = *A* (1 – exp(−*t*/τ)) ignoring the variable initial delay in the current activation [42]. The steady state *I_f_* activation curve was obtained by plotting normalized maximal *I_f_* tail currents at each test potential. The voltage for current half activation (*V*_1/2_) and the slope factor (*s*)were characterized by fitting the activation data to Boltzmann equation [42] using the Clampfit program: *I* = *A*/(1 + exp((*V*_1/2_−*V*)/*s*). The steady state blockade of *I_f_* was defined as 95% of the maximal value. The steady state blockade voltage was calculated by fitting monoexponential equation with the Clampfit program.

### 4.4. ICa,L Measurements

*I_Ca,L_* was measured in whole cell patch clamp mode. Patch pipettes had a resistance of 2–4 MΩ and were filled with solution that contained (in mM) the following: 110 CsCl, 2.5 MgATP, 2.5 Na_2_ATP, 10 HEPES, 5 NaCl, 5 EGTA, 2 CaCl_2_ and 20 TEA-Cl, and titrated to pH 7.2 with CsOH. The extracellular solution was the Tyrode solution as described above. Ten μM tetrodotoxin was added to the Tyrode solution, in the event that interfering currents appeared. Membrane series resistance and whole cell and pipette capacities were routinely compensated electronically up to 90%. Voltage steps ranging from −40 to 40 mV were applied for 300 ms in 5-mV increments from a holding potential of −45 mV (to eliminate interference from I_Ca,T_). The voltage step protocol was applied 3 min after the rupture patch. After IVA application a single step from −45 to 0 mV was applied for 300 ms every 15 s. The voltage steps protocol was applied again 5 min following IVA application. The reductions in I_Ca_ the in range of higher concentrations of IVA are comparable with the results of Bois *et al.* [4], who measured this current in the presence of GTP in the patch pipette. For both cell contraction and electrophysiological recordings, cells from at least five rabbits were used.

### 4.5. Drugs

IVA was obtained from TRC (Toronto, ON, Canada). All other chemicals were purchased from Sigma-Aldrich.

### 4.6. Statistical Analysis

Data are presented as mean ± SEM. For multiple pharmacologic applications, a linear mixed-effects model was used with Dunnett’s method to adjust *p*-values. This model accounts for repeated measurements on the same preparation while allowing testing for differences among different IVA concentrations. *p <* 0.05 was taken to indicate statistical significance. 

## 5. Conclusions

In summary, measuring the relative role of *I_f_* under physiological membrane voltages is required to interpret the results of biophysical measurements and pharmacological effects in physiologically relevant terms and in the context of numerical pacemaker models. The present study shows that the relative IVA blockade of *I_f_* is voltage dependent: at 37 °C in rabbit SANC and at membrane potentials negative to the MDP (unphysiological voltages) both the *I_f_* current amplitude and the effect of IVA to block *I_f_* are higher than at membrane potentials incurred during diastolic depolarization (−65 to −55 mV).
